# Dopamine Development in the Mouse Orbital Prefrontal Cortex Is Protracted and Sensitive to Amphetamine in Adolescence

**DOI:** 10.1523/ENEURO.0372-17.2017

**Published:** 2018-01-10

**Authors:** Daniel Hoops, Lauren M. Reynolds, Jose-Maria Restrepo-Lozano, Cecilia Flores

**Affiliations:** 1 Department of Psychiatry, Douglas Mental Health University Institute, McGill University, Montreal, Quebec H4H 1R3, Canada; 2 Integrated Program in Neuroscience, McGill University, Montreal, Quebec H3A 1A1, Canada

**Keywords:** drug use, guidance cue, orbitofrontal cortex, piriform cortex

## Abstract

The prefrontal cortex (PFC) is divided into subregions, including the medial and orbital prefrontal cortices. Dopamine connectivity in the medial PFC (mPFC) continues to be established throughout adolescence as the result of the continuous growth of axons that innervated the nucleus accumbens (NAcc) prior to adolescence. During this period, dopamine axons remain vulnerable to environmental influences, such as drugs used recreationally by humans. The developmental trajectory of the orbital prefrontal dopamine innervation remains almost completely unstudied. Nonetheless, the orbital PFC (oPFC) is critical for some of the most complex functions of the PFC and is disrupted by drugs of abuse, both in adolescent humans and rodents. Here, we use quantitative neuroanatomy, axon-initiated viral-vector recombination, and pharmacology in mice to determine the spatiotemporal development of the dopamine innervation to the oPFC and its vulnerability to amphetamine in adolescence. We find that dopamine innervation to the oPFC also continues to increase during adolescence and that this increase is due to the growth of new dopamine axons to this region. Furthermore, amphetamine in adolescence dramatically reduces the number of presynaptic sites on oPFC dopamine axons. In contrast, dopamine innervation to the piriform cortex is not protracted across adolescence and is not impacted by amphetamine exposure during adolescence, indicating that dopamine development during adolescence is a uniquely prefrontal phenomenon. This renders these fibers, and the PFC in general, particularly vulnerable to environmental risk factors during adolescence, such as recreational drug use.

## Significance Statement

Dopamine function in the orbital prefrontal cortex (oPFC) underlies many complex cognitive tasks and is disrupted by drugs of abuse. However, the developmental trajectory of the dopamine innervation to this portion of the PFC remains almost completely unstudied. We show that dopamine axons continue to innervate the oPFC during adolescence. This renders these axons particularly vulnerable to environmental influences. Exposure to amphetamine, at doses equivalent to those used recreationally by adolescent people, reduces the number of dopamine connections in the oPFC. This effect is selective; it also occurs in the medial PFC (mPFC) but not in the piriform cortex. The impact of amphetamine on cortical dopamine development in adolescence appears to be a uniquely prefrontal phenomenon.

## Introduction

The prefrontal cortex (PFC) is one of the last brain regions to mature, with changes in structure and connectivity continuing through adolescence and into early adulthood (Paus, 2005; Casey et al., 2008; Caballero et al., 2016). In parallel with these changes, higher order cognitive process that depend on PFC function are also maturing (Sturman and Moghaddam, 2011; Luna et al., 2015; Doremus-Fitzwater and Spear, 2016). Because the function of the PFC is profoundly influenced by the dopamine innervation it receives (Ranganath and Jacob, 2016; Hoops and Flores, 2017), mesocortical dopamine development is an important component of this maturational process. However, the PFC is separated into distinct anatomic regions with distinct functions (Kolb, 2006; Fuster, 2015; Gourley and Taylor, 2016) and our understanding of PFC dopamine development is almost entirely restricted to its medial region (mPFC).

Dopamine connectivity in the mPFC continues to be established throughout adolescence and into early adulthood in both rodents and primates (Kalsbeek et al., 1988; Rosenberg and Lewis, 1995; Benes et al., 2000; Manitt et al., 2011; Naneix et al., 2012; Willing et al., 2017). In mice, the protracted development of the dopamine input to the mPFC results from the ongoing growth of dopamine axons during adolescence (Reynolds et al., 2017). These axons innervate the nucleus accumbens (NAcc) at the onset of adolescence and grow out of the NAcc during the adolescent period. They are uniquely vulnerable to environmental influences during adolescence and exposure to stimulant drugs alters their development, in part by denuding them of presynaptic sites (Reynolds et al., 2015). These alterations result in persistent cognitive changes in adulthood (Reynolds et al., 2015; see also Ye et al., 2014; Kang et al., 2016).

Whether the dopamine innervation to the orbital PFC (oPFC) also continues through adolescence remains understudied (but see Kalsbeek et al., 1988). In rodents and primates, the oPFC plays a central role in some of the most complex cognitive functions of the PFC, including decision-making, cognitive control, and sociality (Bari and Robbins, 2013; Pellis and Pellis, 2013; Wilson et al., 2014). Furthermore, exposure to stimulant drugs alters the structure and function of the oPFC, impacting associated behaviors (Schoenbaum et al., 2006; Goldstein and Volkow, 2011).

There is a longstanding controversy surrounding the designation of the PFC in the rodent and whether it is truly homologous to the PFC in the primate (Preuss, 1995; Uylings et al., 2003; Kolb, 2006; Wise, 2008; Carlén, 2017). However, many characteristics of the PFC important to this study, such as its adolescent maturation (Paus, 2005; Casey et al., 2008; Caballero et al., 2016) and the corresponding changes in cognition and behavior (Sturman and Moghaddam, 2011; Luna et al., 2015; Doremus-Fitzwater and Spear, 2016), are shared between rodents and primates. Furthermore, recent literature supports the hypothesis that the oPFC in particular is homologous between primates and rodents (Heilbronner et al., 2016; Izquierdo, 2017).

In this study, we first characterized the adolescent dopamine innervation to the oPFC in mice. We then examined whether exposure to amphetamine in adolescence affects oPFC dopamine development. To determine if the adolescent trajectory of the dopamine innervation to the PFC is unique, we conducted parallel studies in the adjacent piriform cortex, which is not part of the PFC.

## Materials and Methods

### Animals

Experiments and procedures were performed in accordance with the guidelines of the McGill University animal care committee’s regulations. Mice were received from Charles River Canada and housed with same-sex littermates at the McGill University Neurophenotyping Center on a 12/12 h light/dark cycle with ad libitum access to food and water. In this study we define “early adolescence” in C57/BL6 mice as between the weaning day [postnatal day (PND)21] and PND32 (Hoops and Flores, 2017). Male mice were used for all experiments.

### Drug administration

D-amphetamine sulfate (amphetamine; Sigma-Aldrich) was dissolved in 0.9% saline. Mice were assigned to “drug” and “saline” groups and received one injection of either amphetamine (dose: 4 mg/kg) or saline via intraperitoneal injection once every other day for a total of five injections. This treatment regimen was administered during early adolescence, commencing on PND22 ± 1 and terminating on PND31 ± 1. This regiment has previously been shown to alter mPFC development when administered in early adolescence but not adulthood (Reynolds et al., 2015).

We chose a dose of amphetamine that would achieve a blood plasma concentration in rodents (Riffee et al., 1978; Feldpausch et al., 1998; Van Swearingen et al., 2013) within the range of plasma levels achieved by recreational intake of amphetamine in human adolescents (Kramer et al., 1967; Änggård et al., 1970; Änggård et al., 1970; Gustavsen et al., 2006). Blood plasma concentrations of a lipophilic drug in humans and rodents are likely to result in similar drug concentrations in the brain (Kuczenski and Segal, 2005).

### Tissue processing

Mice were euthanized with an intraperitoneal injection of 50 mg/kg ketamine, 5 mg/kg xylazine, and 1 mg/kg acepromazine. They were then perfused intracardially with 10 IU/ml heparinized saline followed by 4% paraformaldehyde. Both perfused solutions were pH adjusted to between 7.2 and 7.4 with dilute hydrochloric acid and sodium hydroxide. After perfusion, brains were dissected from the skull, placed in 4% paraformaldehyde overnight at 4°C, and then stored in phosphate-buffered saline at 4°C. After up to 2 d in storage, brains were cut coronally into 35-μm-thick sections on a vibratome.

Every second section was processed for immunofluorescence. A polyclonal rabbit anti-tyrosine hydroxylase (TH; a commonly used marker for dopamine) antibody (Millipore product #AB152, RRID #AB_390204, 1:1000 dilution) and an Alexa Fluor 594-conjugated polyclonal donkey anti-rabbit antibody (The Jackson Laboratory product #711585152, RRID #AB_2340621, 1:500 dilution) were used as primary and secondary antibodies. The manufacturer’s certificate of analysis reports that the immunogen for the primary antibody is denatured TH from rat pheochromocytoma. Its specificity has been verified by Western blotting (Millipore) and immunoprecipitation (Haycock, 1987). The fibers labeled by this antibody show features indistinguishable from the classic features of cortical dopamine axons in rodents (Berger et al., 1974, 1983; Van Eden et al., 1987; Manitt et al., 2011), namely they are thin fibers with irregularly-spaced varicosities, increase in density toward the deep cortical layers, and are not regularly oriented in relation to the pial surface. This is in contrast to rodent norepinephrine fibers, which are smooth or beaded in appearance, relatively thick with regularly spaced varicosities, increase in density toward the shallow cortical layers, and are in large part oriented either parallel or perpendicular to the pial surface (Berger et al., 1974, 1983; Levitt and Moore, 1979; Miner et al., 2003). Furthermore, previous studies in rodents have noted that only norepinephrine cell bodies are detectable using immunofluorescence for TH, not norepinephrine processes (Pickel et al., 1975; Verney et al., 1982; Miner et al., 2003), and we did not observe any norepinephrine-like fibers. After immunofluorescence staining, sections were mounted onto gel-coated slides and cover-slipped with a DAPI-containing hardset mounting medium (product #H1500, Vector Laboratories).

### Axon tracking

To prove conclusively that the protracted dopamine innervation to the oPFC during adolescence is due to new axon growth and not the branching of fibers that reached the oPFC earlier in life, we adapted an axon-initiated viral transduction technique (Beier et al., 2015) to track the growth of dopamine axons in adolescent mice. Briefly, at PND22 a retrogradely transported virus expressing Cre recombinase (CAV-Cre BioCampus Montpellier; Bru et al., 2010) was injected unilaterally at the level of the NAcc. A Cre-dependent enhanced yellow fluorescent protein (eYFP) virus DIO-eYFP (pAAV-Ef1a-DIO-EYFP-WPRE-pA, UNC Vector Core) was simultaneously injected into the ipsilateral ventral tegmental area. CAV-Cre requires the coxsackievirus adenovirus receptor to be taken up and is therefore preferentially taken up by axon terminals (Bru et al., 2010). This recombination strategy limits eYFP labeling to ventral tegmental area neurons with axons that terminate in the NAcc at PND22. Dopamine axons that continue to grow from the NAcc to the oPFC in adolescence can be detected in adulthood by stereologically counting eYFP-expressing dopamine varicosities in the oPFC. To rule out the possibility of detecting dopamine axons in the NAcc that send collaterals to the oPFC, identical dual viral injections were performed in three adult mice, and we counted eYFP-expressing dopamine varicosities in the oPFC three months later. It is important to note that this virally-mediated axon-tracking technique only infects a small percentage of ventral tegmental area neurons that innervate the NAcc (Beier et al., 2015). Therefore, the number of TH-immunolabelled (TH+) varicosities in the adult oPFC that also express eYFP (eYFP+) is an underestimation of the total population of dopamine varicosities on axons that have grown to the oPFC during adolescence.

### Stereotaxic surgery

Early adolescent (PND22 ± 1) wild-type mice were anesthetized with isoflurane. Simultaneous unilateral stereotaxic infusions of DIO-eYFP into the VTA and CAV-Cre into the NAcc were performed using Hamilton syringe needles. Coordinates were previously verified by viral and/or dye injection (Manitt et al., 2013; Reynolds et al., 2017). The coordinates we used were: VTA: -2.56 mm (anterior/posterior), +0.9 mm (lateral), and -4.1 mm (dorsal/ventral) relative to bregma, at a 10° angle; and NAcc: +2.6 mm (anterior/posterior), +1.5 mm (lateral), and -3.75 mm (dorsal/ventral) relative to bregma, at a 30° angle. A total of 0.5 μl of purified virus was delivered over an 8-min period followed by a pause of 6 min. Adult (PND75 ± 15) wild-type mice underwent the same injection procedure using the following coordinates: VTA: -3.2 mm (anterior/posterior), +1.0 mm (lateral), and -4.6 mm (dorsal/ventral) relative to bregma, at a 10° angle; NAcc: +1.8 mm (anterior/posterior), +3.0 mm (lateral), and -4.8 mm (dorsal/ventral) relative to bregma, at a 30° angle (Manitt et al., 2013; Daubaras et al., 2014). Carpofen was delivered subcutaneously during surgery and as a diet supplement (MediGel CPF, Clear H_2_O) throughout recovery for pain management.

### Stereological analyses

Contours of oPFC subregions and the piriform cortex were delineated on sections corresponding to plates 14–20 of the mouse brain atlas, resulting in delineations on five sections per region per mouse (Paxinos and Franklin, 2013). We delineated four oPFC subregions: the dorsal agranular insular PFC (daiPFC), the ventral agranular insular PFC (vaiPFC), the lateral oPFC (loPFC), and the ventral oPFC (voPFC) using landmarks derived from Paxinos and Franklin (2013). While the oPFC is present at more anterior levels of the mouse brain, dopamine innervation at these levels is practically absent. The medial subregion of the oPFC is only present at these anterior levels and therefore is not included in our study.

Previous work in the mPFC that uses similar methods delineates only the dense dopamine innervation present in the deep cortical layers (Manitt et al., 2011, 2013; Reynolds et al., 2015). However, the dopamine innervation to the oPFC is diffuse compared to the mPFC and no clearly demarcated area of dopamine innervation is present. Therefore, in this study we only estimate the density of dopamine varicosities within each oPFC subregion; we did not measure the span of the dopamine innervation as we have done previously (Manitt et al., 2011, 2013).

Each cortical subregion was traced at x 5 magnification with a Leica DM400B microscope and Stereoinvestigator software (MicroBrightField). To estimate the number of labeled varicosities, we used the optical fractionator probe function of Stereoinvestigator. We counted TH+ and eYFP+ varicosities, as well as double-labeled varicosities, as appropriate for each experiment. Varicosities were defined as dilated elements associated with axonal processes and were thus only counted if they were clearly associated with an axon (Parish et al., 2002; Manitt et al., 2011). A grid of 175 μm^2^ was superimposed on each contour, starting at a random point within the contour. Unbiased counting frames (25 μm^2^) were placed in the top left corner of each grid square. Depending on the area of the region of interest, between 10 and 35 counting frames were quantified per delineation. Counting was conducted at 100× magnification on every other mounted section using a counting depth of 10 μm and a guard zone of 5 μm. Section thickness was measured while counting and counts were performed blind by a single individual. To assess the volume of each delineated region we used the Cavalieri volume estimation method in Stereoinvestigator, with a grid of 100 μm^2^. To calculate varicosity density for each region of interest in each brain, we divided the total varicosity estimates obtained using the optical fractionator by the estimated volume obtained using the Cavalieri estimator.

Varicosities were used as the counting unit to obtain a measure of dopamine presynaptic density because nearly every dopamine varicosity in the PFC forms a synapse (Séguéla et al., 1988). Varicosities also represent sites where neurotransmitter synthesis, packaging, release, and reuptake most often occur (Benes et al., 1996). As is standard practice for our group, we obtained counts only from the right hemisphere.

### Statistical analyses

To analyze varicosity density, we conducted two-way mixed ANOVAs with oPFC subregion and either age (adolescent and adult, result 3.1) or treatment (saline or amphetamine, result 3.3) as fixed factors, mouse as the random factor, and varicosity density as the response variable. Each age and treatment group had a sample size of four mice. Subsequently, we conducted Student’s *t* tests, corrected for multiple comparisons using the Hochberg-Bonferroni procedure (Hochberg, 1988), between treatment groups separately for each oPFC subregion in all cases where either subregion or the interaction term was significant in the ANOVA. We analyzed data for the piriform cortex independently using Student’s *t* tests. All data used in this study are available as extended data.

**Figure 1.**
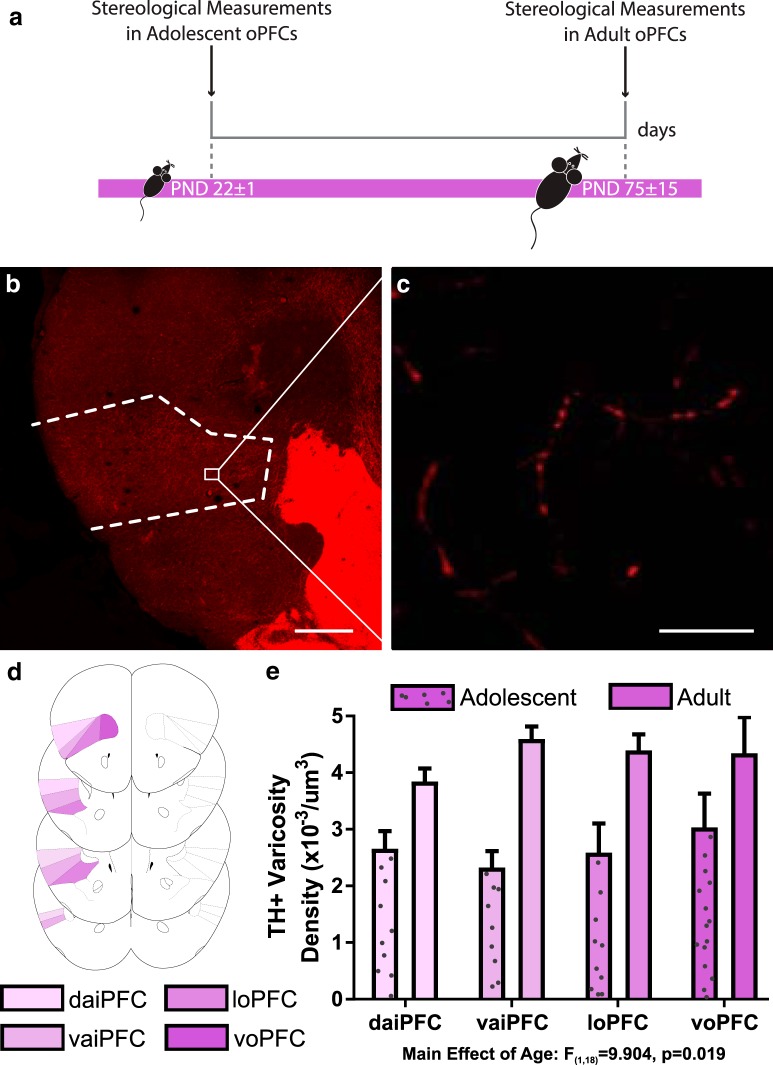
Dopamine varicosity density in the oPFC is protracted across adolescence. ***A***, Timeline of experimental procedures; *n* = 4 per group. ***B***, A micrograph of a coronal section through the frontal cortex of an adult mouse at low magnification (4×) showing the contour of the oPFC. Scale bar = 500 μm. ***C***, A micrograph of a coronal section of the oPFC of an adult mouse at high magnification (60×) showing TH-immunopositive varicosities. Scale bar = 10 μm. ***D***, The voPFC, loPFC, vaiPFC, and daiPFC respectively, are highlighted in increasingly pale shades of purple. Line drawings were derived from Paxinos and Franklin (2013). ***E***, Stereological quantification of dopamine varicosity density reveals that there are more dopamine varicosities in the adult oPFC than the adolescent. Bars represent mean ± standard error (Extended Data >Fig. 1-1).

**Figure 2.**
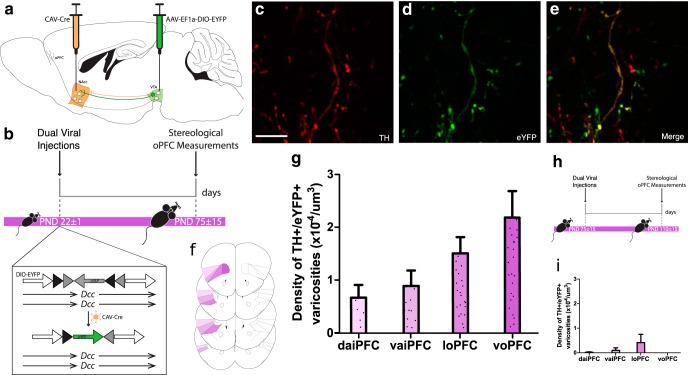
Axons continue to grow to the oPFC during adolescence. ***A***, The dual-viral injection method used to label NAcc-projecting ventral tegmental area neurons with eYFP. ***B***, Timeline of experimental procedures: mice were injected at the start of adolescence (PND22 ± 1) and six weeks later, at which point adolescent mice have reached adulthood, eYFP-expressing dopamine axons in the oPFC were quantified; *n* = 5. ***C–E***, Micrographs of a coronal section of the oPFC of an adult mouse at high magnification (60×) show (***C***) TH-immunopositive varicosities, (***D***) eYFP-expressing varicosities, and (***E***) an overlay highlighting co-labeled varicosities. Scale bar = 10 μm. ***F***, The voPFC, loPFC, vaiPFC, and daiPFC, respectively, are highlighted in increasingly pale shades of purple. Line drawings were derived from Paxinos and Franklin (2013). ***G***, Stereological quantification of dopamine varicosity density reveals eYFP-expressing dopamine varicosities are present in the oPFC in adult mice that received dual-viral injections in early adolescence (Extended Data Fig. 2-1). ***H***, To ensure the eYFP-expressing dopamine neurons in the oPFC were the result of axon growth and not collaterals, we injected viruses into early adult mice and quantified eYFP-expressing dopamine axons six weeks later; *n* = 3. ***I***, eYFP-expressing dopamine varicosities are almost entirely absent from the orbital prefrontal cortices of mice that were injected during adulthood (Extended Data Fig. 2-1). **G & I**, Bars represent mean ± standard error.

**Figure 3.**
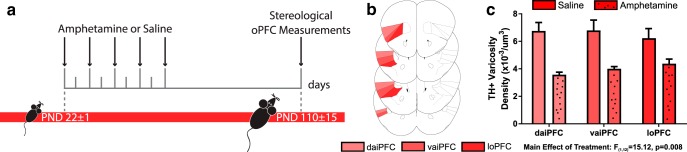
Amphetamine in adolescence alters dopamine connectivity in adulthood. ***A***, Timeline of experimental procedures; *n* = 4 per group. ***B***, The loPFC, vaiPFC, and daiPFC, respectively, are highlighted in increasingly pale shades of red. Line drawings were derived from Paxinos and Franklin (2013). ***C***, Stereological quantification of dopamine varicosity density reveals that adults exposed to amphetamine during adolescence have about a 40% reduction in dopamine varicosity density in the oPFC compared to saline-treated controls. Bars represent mean ± standard error (Extended Data Fig. 3-1).

**Figure 4.**
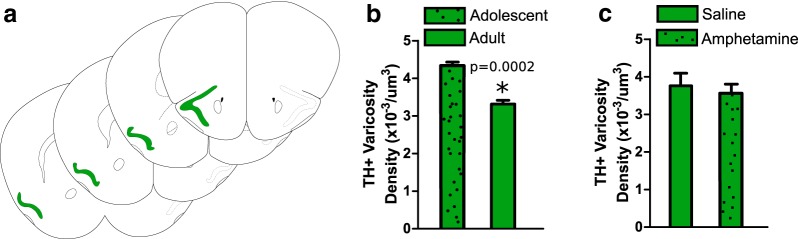
Dopamine connectivity in the piriform cortex is not protracted nor influenced by amphetamine in adolescence. ***A***, The piriform cortex was outlined based on Paxinos and Franklin (2013) and is highlighted in green. ***B***, ***C***, Stereological quantification of dopamine varicosity density reveals that (***B***) early adolescent mice have a significantly higher density of dopamine varicosities in the piriform cortex compared to adults (Extended Data Fig. 4-1) and (***C***) that amphetamine exposure during adolescence does not alter the density of dopamine varicosities in the piriform cortex. Bars represent mean ± standard error (Extended Data Fig. 4-2).

10.1523/ENEURO.0372-17.2017.ed1 Extended Data Legends **Extended Data Figure 1-1.** Dopamine (TH-immunopositive) varicosity density estimates for four subregions of the oPFC in adult and early adolescent mice. Varicosity density was estimated by combining the optical fractionator and Cavelieri estimator methods of the software program Stereoinvestigator (see Materials and Methods). These data were used to generate the results presented in Results, Dopamine innervation to the oPFC is protracted across adolescence, and Figure 1.
**Extended Data Figure 2-1.** Density estimates of dopamine (TH; TH-immunopositive) varicosities that have been infected with a fluorescent protein-expressing virus for four subregions of the oPFC in adult and early adolescent mice. Varicosity density was estimated by combining the optical fractionator and Cavelieri estimator methods of the software program Stereoinvestigator (see Materials and Methods). These data were used to generate the results presented in Results, Delayed dopamine innervation to the oPFC results from ongoing axon growth, and Figure 2.
**Extended Data Figure 3-1.** Dopamine (TH-immunopositive) varicosity density estimates in four subregions of the oPFC in adult mice treated with either saline or amphetamine in early adolescence. Varicosity density was estimated by combining the optical fractionator and Cavelieri estimator methods of the software program Stereoinvestigator (see Materials and Methods). These data were used to generate the results presented in Results, Amphetamine in adolescence reduces dopamine varicosity density in the adult oPFC, and Figure 3.
**>Extended Data Figure 4-1.** Dopamine (TH-immunopositive) varicosity density estimates for the piriform cortex in adult and early adolescent mice. Varicosity density was estimated by combining the optical fractionator and Cavelieri estimator methods of the software program Stereoinvestigator (see Materials and Methods). These data were used to generate the results presented in Results, Dopamine innervation to the piriform cortex is not protracted nor influenced by amphetamine in adolescence, and Figure 4*B*.
**Extended Data Figure 4-2.** Dopamine (TH-immunopositive) varicosity density estimates for the piriform cortex in adult mice treated with either saline or amphetamine in early adolescence. Varicosity density was estimated by combining the optical fractionator and Cavelieri estimator methods of the software program Stereoinvestigator (see Materials and Methods). These data were used to generate the results presented in Results, Dopamine innervation to the piriform cortex is not protracted nor influenced by amphetamine in adolescence, and Figure 4*C*. Download Tables, DOCX file.

## Results

### Dopamine innervation to the oPFC is protracted across adolescence

We found that the density of dopamine varicosities in the oPFC is significantly higher in adult mice compared to adolescent mice, indicating that dopamine innervation continues to increase in the oPFC during adolescence (Fig. 1; Extended Data Fig. 1-1; two-way ANOVA, main effect of age, *F*_(1,18)_ = 9.904, *p* = 0.019, no significant effect of region, *F*_(3,18)_ = 1.30, *p* = 0.305, or age × region interaction, *F*_(3,18)_ = 2.47, *p* = 0.095). *Post hoc* Hochberg-corrected *t* tests revealed that dopamine innervation increases during adolescence in the loPFC (*t*_(6)_ = 3.091, *p* = 0.021), daiPFC (*t*_(6)_ = 3.062, *p* = 0.022), and vaiPFC (*t*_(6)_ = 6.282, *p* = 0.0008), but not in the voPFC (*t*_(6)_ = 1.495, *p* = 0.186). Visual comparison of densities between adolescence and adulthood in the voPFC (Fig. 1*E*) indicates that high within-group variability underlies the nonsignificant *p* value in the voPFC.

### Delayed dopamine innervation to the oPFC results from ongoing axon growth

The axon-initiated recombination technique we used limits eYFP expression to ventral tegmental area neurons with axons that have reached the NAcc by the start of adolescence (Beier et al., 2015; Reynolds et al., 2017). If the axons of these neurons continue to grow to the oPFC during adolescence, we should observe eYFP-positive dopamine varicosities in the adult oPFC. Remarkably, we find eYFP dopamine varicosities in all four oPFC subregions in adult mice that received dual viral infection in early adolescence (Fig. 2; Extended Data Fig. 2-1). The presence of these varicosities indicates that dopamine axons indeed continue to grow to the oPFC from the NAcc during adolescence. To exclude the possibility that these eYFP-positive dopamine varicosities are collaterals of fibers innervating the NAcc, we performed the same axon-initiated viral tracing experiment in adult mice. eYFP-positive dopamine varicosities in the oPFC are absent or negligible in these mice (Fig. 2; Extended Data Fig. 2-1).

### Amphetamine in adolescence reduces dopamine varicosity density in the adult oPFC

Amphetamine in adolescence has been found to reduce the number of presynaptic sites from mPFC dopamine axons, measured as a significant reduction in dopamine varicosity density (Reynolds et al., 2015). Here, we measured the density of dopamine varicosities in the oPFC in adult mice exposed to the exact same regimen of amphetamine or saline in adolescence as in Reynolds et al. (2015). Similar to the mPFC, amphetamine in adolescence results in significantly fewer dopamine varicosities in the oPFC (Fig. 3; Extended Data Fig. 3-1; two-way ANOVA, main effect of age, *F*_(1,12)_ = 15.12, *p* = 0.008, no significant effect of region, *F*_(2,12)_ = 0.42, *p* = 0.670, or age × region interaction, *F*_(2,12)_ = 3.68, *p* = 0.057). *Post hoc* Hochberg-corrected *t* tests revealed that dopamine varicosities were reduced in the daiPFC (*t*_(6)_ = 4.987, *p* = 0.003) and vaiPFC (*t*_(6)_ = 3.630, *p* = 0.011), while the effect in the loPFC was marginal (*t*_(6)_ = 2.370, *p* = 0.056).

### Dopamine innervation to the piriform cortex is not protracted nor influenced by amphetamine in adolescence

We measured dopamine varicosity density in the piriform cortex, a cortical region that is not part of the PFC, in adolescent and adult mice. In contrast to the mPFC (Kalsbeek et al., 1988) and oPFC, dopamine varicosity density in the piriform cortex is significantly higher in adolescence compared to adulthood (Fig. 4; Extended Data Fig. 4-1; *t*_(6)_ = 8.256, *p* = 0.0002).

In addition, amphetamine during adolescence does not alter dopamine varicosity density: levels are similar between adult mice treated with drug or saline in adolescence (Fig. 4; Extended Data Fig. 4-2; *t*_(6)_ = 0.7985, *p* = 0.455). This indicates that it is specifically the dopamine innervation to the PFC that is sensitive to amphetamine during adolescence.

## Discussion

The increase in mPFC dopamine innervation during adolescence is a well-known phenomenon, both in rodents and primates (Kalsbeek et al., 1988; Rosenberg and Lewis, 1995; Benes et al., 2000; Manitt et al., 2011; Naneix et al., 2012; Reynolds et al., 2017; Willing et al., 2017). However, until now adolescent dopamine innervation in the oPFC remained almost completely unstudied (but see Kalsbeek et al., 1988). Here, we show that in mice the dopamine innervation to the oPFC is still maturing during adolescence. Specifically, oPFC dopamine varicosity density increases during this period. Critically, this increase is due to the growth of new axons to the oPFC. Because oPFC dopamine axons are still growing, they remain vulnerable to environmental influences for an extended spatiotemporal window. We find that exposure to amphetamine during adolescence reduces the number of dopamine varicosities in the oPFC in adulthood. Notably, these effects appear to be specific to the PFC; we do not find them in the adjacent piriform cortex.

Although we discuss our findings in the context of the relevant literature, we note that the matter of PFC homology between the rodent and primate is not yet settled (Carlén, 2017). However, available evidence strongly supports homology between the rodent and primate oPFC (Uylings et al., 2003; Heilbronner et al., 2016; Izquierdo, 2017). Finally, homology between the primate and rodent oPFC would imply that the oPFC is homologous among rodents, however to our knowledge there are no studies directly examining this. Therefore, we encourage caution in translating our findings to other rodents, including rats.

The Netrin-1 receptor DCC (deleted in colorectal cancer) is responsible for coordinating dopamine axon structure and function in the mPFC (Hoops and Flores, 2017). Reducing DCC expression on ventral tegmental neurons in adolescence induces structural deficiencies in mPFC dopamine axons (Reynolds et al., 2017), and amphetamine in adolescence reduces DCC expression on these neurons (Yetnikoff et al., 2010, 2011). These alterations result in persistent cognitive changes in adulthood (Reynolds et al., 2015). Our results suggest that amphetamine in adolescence is reducing DCC expression on ventral tegmental dopamine neurons that project to the oPFC, resulting in denuded dopamine axons in this region. Furthermore, since the vast majority of dopamine varicosities form synapses (Séguéla et al., 1988), the reduction we see in varicosities in amphetamine-treated mice suggests that dopamine axons are forming far fewer synapses in the oPFC as compared to mice treated with saline. Therefore, our results suggest that the growing dopamine axons are vulnerable to environmental influences and that amphetamine exposure during adolescence may impair the formation of dopamine synapses onto their target cells within the oPFC.

The piriform cortex, part of the mammalian olfactory cortex, is structurally and functionally linked to the oPFC in rodents and humans (Gottfried et al., 2003; Uylings et al., 2003; Fuster, 2015) and in rodents receives about the same density of dopamine input as the oPFC (Fallon and Loughlin, 1987). However, unlike the oPFC, the piriform cortex matures relatively early in human postnatal development (Gogtay et al., 2004; Shaw et al., 2008), as do piriform cortex-dependent olfactory discrimination behaviors in rodents (Staubli et al., 1987; Hu et al., 1997). Therefore, the piriform cortex was an ideal cortical region to use to examine whether our results are specific to the PFC or reflect general trends in frontal cortex development. To our knowledge, this is the first time that the postnatal developmental time course of the dopamine innervation to the piriform cortex has been studied, and so we feel that our findings in this region are also important in their own right. We find that adolescent mice had a higher density of dopamine varicosities compared to adult mice, the opposite pattern to the one we observed in the oPFC. However, this result is in line with what is observed in the cortex as a whole, where there is an overproduction of synapses during early adolescence followed by a period of rapid pruning in primates and rodents (Lidow et al., 1991; Andersen, 2003; Crews et al., 2007; Juraska and Willing, 2017). In fact, it has been estimated that in primates as many as 30 000 synapses may be lost per second over the entire cortex during adolescence, resulting in an overall loss of up to one half the total number of synapses present during early adolescence (Rakic et al., 1994). Our findings are also in line with a study that found dopamine receptor expression in the rat piriform cortex peaks in adolescence before declining in adulthood (Garske et al., 2013). Therefore, axon growth and synapse formation during adolescence appears to be a unique characteristic of the mesocortical dopamine projections to the PFC.

## Conclusion

Taken together, our results show that dopamine development to the oPFC is delayed across adolescence. Importantly, we have found that this delayed growth is due to axons that previously innervated the NAcc leaving this region and growing to the oPFC during this period. Furthermore, we show that these axons are vulnerable to environmental influences such as exposure to addictive drugs. Amphetamine exposure during early adolescence results in reduced synapse formation in the oPFC. Finally, dopamine innervation to the piriform cortex reaches adult densities before adolescence, indicating that delayed growth of dopamine axons, and the associated vulnerability to environmental influences, is specific to the PFC.

## Synthesis

Reviewing Editor: Karen Szumlinski, University of California at Santa Barbara Decisions are customarily a result of the Reviewing Editor and the peer reviewers coming together and discussing their recommendations until a consensus is reached. When revisions are invited, a fact-based synthesis statement explaining their decision and outlining what is needed to prepare a revision will be listed below. The following reviewer(s) agreed to reveal their identity: David Moorman.

Following consultation with two expert reviewers, we have come to the decision of “Revise and Re-review” for your article. This decision was based on the enthusiasm we all have for the significance of the topic of study, but concerns over the Methods and Results as currently written. None of the revisions should require more than two months' work. The specific comments of the reviewers are listed below:

Reviewer 1:

Major:

I take issue with description throughout as 'prefrontal cortex' and the loose/interchangeable citation of primate and rodent literature. Rodents do not have a granular PFC. There is indeed controversy about homology (see Preuss, 1995; Wise, 2008) though OFC may be safer to make such claims as being within PFC. For example, connectivity-based inferences about OFC-striatum homology between primate and rodent are supported by recent literature (see Heilbronner et al. Biol Psychiatry 2016 and recent commentary by Izquierdo, 2017 in J Neurosci). Suggest referring to as frontal cortex or OFC, or to make much more clear as referring to 'mouse orbitofrontal cortex.'

Similarly, the title should be more informative that this is mouse orbitofrontal cortex that is being studied.

There is poor detail about surgical procedures in the young animals. Were there special stereotaxic procedures for an animal of that size? There are no details on anesthesia, analgesics, for the axon tracking surgeries where virus is injected in the brain. Please provide.

The effects reported here are more pronounced in certain subregions of OFC over others, an interesting finding that is not emphasized, and may speak to functional heterogeneity within rodent OFC (Izquierdo, 2017). For example, authors found that dopamine innervation increases during adolescence in the lateral OFC, dorsal AI, and ventral AI, but not ventral OFC. Authors maintain that this is because ventral OFC is located adjacent to the dorsal striatum and that fibers from NAcc to OFC run anteriodorsally through dorsal striatum and forceps minor. Is there any other anatomical evidence of this proposed mechanism? Please clarify. Additionally, it is interesting that dopamine innervation at more anterior levels (and consequently medial subregion) is very low/absent. It is not clear to me what the authors make of it; there may be functional (behavioral) consequences following developmental amphetamine exposure because of this, but it is not discussed.

Lines 265-266 “until now adolescent dopamine development in the oPFC remained almost completely unstudied...” This seems overstated since different groups have looked at other markers of dopamine development, such as dopamine receptor density and expression in rodent frontal cortex in adolescent development (Tarazi & Baldessarini, 2000; Garske et al. 2013).

Some mention of how much of these findings in mouse may be translated to rat (not just primate) would also be welcome.

Minor comments:

Lines 33-34 of Abstract- do the authors mean to say instead that amphetamine impacts dopamine development in adolescence uniquely in PFC?

Lines 95-97- the dosing regimen of amphetamine was chosen to achieve a blood plasma concentration in rodents that is similar to that of recreational intake of amphetamine in human adolescents. What is this dose? and was it confirmed here in the present study via blood collection?

Lines 313-315, “this is the first time that the postnatal developmental time course of the dopamine projection to the piriform cortex has been studied.” But authors later cite Garske et al. 2013 that studied OFC and piriform cortex in adolescence.

Line 91, regimen

Reviewer 2:

1. The phrasing of “growth of dopamine axons from the nucleus accumbens” or a variant is used throughout the manuscript. This is confusing because it sounds like the authors are saying that dopamine, originating in the NAcc, is going to the OFC. This should be changed to clarify.

2. The authors argue that TH staining in OFC is not due to noradrenergic inputs from the locus coeruleus. They cite a number of papers showing limited colocalization between NET and TH, indicating that TH staining primarily identifies dopaminergic axons. However, at least one cited paper (Verney et al., 1982) discuss a population of TH-immunoreactive fibers that disappear after lesioning of the locus coeruleus, indicating that there may be some detection of noradrenergic fibers. This should be considered thoroughly as the manuscript depends on TH immunohistochemistry reflecting dopamine.

3. Figure 1D should show regions analyzed in a color-coded fashion. I also don't see where VO is marked on the anatomy figure. The specific details of anatomical demarcations could be cleared up.

4. Can CAV enter axons through en passant boutons? If so, is there any concern that the use of CAV in the NAcc is labeling fibers that project through the NAcc?

5. In the discussion, the authors state that adult levels were reached in voPFC prior to the onset of adolescence. This isn't clear from figure 1, where the difference between adolescent and adult density is similar in voPFC the other regions, and that there is an increase between adolescence and adults. Is this a real difference or an artifact of statistical analysis on very small numbers?

6. Related to 5, there is a decent amount of speculation in the discussion about why there is no statistical difference in voPFC. None of this is based on much evidence. The finding that there are TH+/eYFP+ fibers in adult voPFC as supporting evidence was confusing to me.

7. Is there a linear relationship between dopamine varicosities and axons? If so, this should be noted somewhere (unless I missed it). If not, much of the framing of the discussion should be rewritten, given that the study measured varicosities and the authors use this information to make claims about axonal presence and structure.

8. Is there a significant difference between saline treated animals in figure 3C and adult animals in figure 1D? There are differences, and if any of these are significant, it potentially undermines the intended comparisons (adolescent vs. adult or saline vs. amphetamine), given that all comparisons are across-subject.
