# Supplementary material for: Dopamine Development in the Mouse Orbital Prefrontal Cortex Is Protracted and Sensitive to Amphetamine in Adolescence
Source: eNeuro. 2018 Jan 10;5(1):ENEURO.0372-17.2017. doi: 10.1523/ENEURO.0372-17.2017 (PMC5762649; doi:10.1523/ENEURO.0372-17.2017)
Supplement: Extended Data Legends — Extended Data Figure 1-1. Dopamine (TH-immunopositive) varicosity density estimates for four subregions of the oPFC in adult and early adolescent mice. Varicosity density was estimated by combining the optical fractionator and Cavelieri estimator methods of the software program Stereoinvestigator (see Materials and Methods). These data were used to generate the results presented in Results, Dopamine innervation to the oPFC is protracted across adolescence, and Figure 1. Extended Data Figure 2-1. Density estimates of dopamine (TH; TH-immunopositive) varicosities that have been infected with a fluorescent protein-expressing virus for four subregions of the oPFC in adult and early adolescent mice. Varicosity density was estimated by combining the optical fractionator and Cavelieri estimator methods of the software program Stereoinvestigator (see Materials and Methods). These data were used to generate the results presented in Results, Delayed dopamine innervation to the oPFC results from ongoing axon growth, and Figure 2. Extended Data Figure 3-1. Dopamine (TH-immunopositive) varicosity density estimates in four subregions of the oPFC in adult mice treated with either saline or amphetamine in early adolescence. Varicosity density was estimated by combining the optical fractionator and Cavelieri estimator methods of the software program Stereoinvestigator (see Materials and Methods). These data were used to generate the results presented in Results, Amphetamine in adolescence reduces dopamine varicosity density in the adult oPFC, and Figure 3. >Extended Data Figure 4-1. Dopamine (TH-immunopositive) varicosity density estimates for the piriform cortex in adult and early adolescent mice. Varicosity density was estimated by combining the optical fractionator and Cavelieri estimator methods of the software program Stereoinvestigator (see Materials and Methods). These data were used to generate the results presented in Results, Dopamine innervation to the piriform cortex is [file sup_enu-eN-NWR-0372-17-s02.docx]

**Extended Data**

**Figure 1-1.** Data used to generate panel 1e.

| **Mouse** | **Age** | **Region** | **Dopamine Varicosity Density (counts/um^3)** |
| --- | --- | --- | --- |
| Adol1of4 | Adolescent | daiPFC | 0.00338 |
| Adol1of4 | Adolescent | vaiPFC | 0.00296 |
| Adol1of4 | Adolescent | loPFC | 0.00391 |
| Adol1of4 | Adolescent | voPFC | 0.00478 |
| Adol2of4 | Adolescent | daiPFC | 0.00297 |
| Adol2of4 | Adolescent | vaiPFC | 0.00267 |
| Adol2of4 | Adolescent | loPFC | 0.00286 |
| Adol2of4 | Adolescent | voPFC | 0.00280 |
| Adol3of4 | Adolescent | daiPFC | 0.00203 |
| Adol3of4 | Adolescent | vaiPFC | 0.00187 |
| Adol3of4 | Adolescent | loPFC | 0.00198 |
| Adol3of4 | Adolescent | voPFC | 0.00249 |
| Adol4of4 | Adolescent | daiPFC | 0.00225 |
| Adol4of4 | Adolescent | vaiPFC | 0.00181 |
| Adol4of4 | Adolescent | loPFC | 0.00161 |
| Adol4of4 | Adolescent | voPFC | 0.00209 |
| Ad3of6 | Adult | daiPFC | 0.00368 |
| Ad3of6 | Adult | vaiPFC | 0.00435 |
| Ad3of6 | Adult | loPFC | 0.00365 |
| Ad3of6 | Adult | voPFC | 0.00381 |
| Ad4of6 | Adult | loPFC | 0.00455 |
| Ad4of6 | Adult | voPFC | 0.00471 |
| Ad4of6 | Adult | daiPFC | 0.00409 |
| Ad4of6 | Adult | vaiPFC | 0.00502 |
| Ad5of6 | Adult | loPFC | 0.00438 |
| Ad5of6 | Adult | voPFC | 0.00294 |
| Ad5of6 | Adult | daiPFC | 0.00329 |
| Ad5of6 | Adult | vaiPFC | 0.00411 |
| Ad7of6 | Adult | daiPFC | 0.00432 |
| Ad7of6 | Adult | vaiPFC | 0.00491 |
| Ad7of6 | Adult | loPFC | 0.00500 |
| Ad7of6 | Adult | voPFC | 0.00592 |

**Figure 2-1.** Data used to generate panels 2f and 2h.

| **Mouse** | **Age** | **Region** | **TH+/eYFP+ Varicosity Density (counts/um^3)** |
| --- | --- | --- | --- |
| 10-8-2. | Adolescent | daiPFC | 0.00015 |
| 10-8-2. | Adolescent | vaiPFC | 0.00019 |
| 10-8-2. | Adolescent | loPFC | 0.00025 |
| 10-8-2. | Adolescent | voPFC | 0.00031 |
| 57-1. | Adolescent | daiPFC | 0.00002 |
| 57-1. | Adolescent | vaiPFC | 0.00003 |
| 57-1. | Adolescent | loPFC | 0.00007 |
| 57-1. | Adolescent | voPFC | 0.00023 |
| 82-1. | Adolescent | daiPFC | 0.00002 |
| 82-1. | Adolescent | vaiPFC | 0.00010 |
| 82-1. | Adolescent | loPFC | 0.00018 |
| 82-1. | Adolescent | voPFC | 0.00003 |
| 82-2. | Adolescent | daiPFC | 0.00010 |
| 82-2. | Adolescent | vaiPFC | 0.00004 |
| 82-2. | Adolescent | loPFC | 0.00016 |
| 82-2. | Adolescent | voPFC | 0.00027 |
| 95-1. | Adolescent | daiPFC | 0.00004 |
| 95-1. | Adolescent | vaiPFC | 0.00007 |
| 95-1. | Adolescent | loPFC | 0.00010 |
| 95-1. | Adolescent | voPFC | 0.00025 |
| 2-2. | Adult | daiPFC | 0.00000 |
| 2-2. | Adult | vaiPFC | 0.00000 |
| 2-2. | Adult | loPFC | 0.00003 |
| 3-3. | Adult | daiPFC | 0.00000 |
| 3-3. | Adult | vaiPFC | 0.00000 |
| 3-3. | Adult | loPFC | 0.00000 |
| 52-8. | Adult | daiPFC | 0.00001 |
| 52-8. | Adult | vaiPFC | 0.00004 |
| 52-8. | Adult | loPFC | 0.00014 |

**Figure 3-1.** Data used to generate panel 3c.

| **Mouse** | **Treatment** | **Region** | **Dopamine Varicosity Density (counts/um^3)** |
| --- | --- | --- | --- |
| 56-1. | Amphetamine | daiPFC | 0.00380 |
| 56-1. | Amphetamine | vaiPFC | 0.00418 |
| 56-1. | Amphetamine | loPFC | 0.00382 |
| 56-2. | Amphetamine | daiPFC | 0.00354 |
| 56-2. | Amphetamine | vaiPFC | 0.00427 |
| 56-2. | Amphetamine | loPFC | 0.00533 |
| 60-1. | Amphetamine | daiPFC | 0.00389 |
| 60-1. | Amphetamine | vaiPFC | 0.00353 |
| 60-1. | Amphetamine | loPFC | 0.00414 |
| 68-2. | Amphetamine | daiPFC | 0.00309 |
| 68-2. | Amphetamine | vaiPFC | 0.00403 |
| 68-2. | Amphetamine | loPFC | 0.00423 |
| 55-1. | Saline | daiPFC | 0.00639 |
| 55-1. | Saline | vaiPFC | 0.00572 |
| 55-1. | Saline | loPFC | 0.00507 |
| 55-2. | Saline | daiPFC | 0.00807 |
| 55-2. | Saline | vaiPFC | 0.00759 |
| 55-2. | Saline | loPFC | 0.00793 |
| 59-1. | Saline | daiPFC | 0.00524 |
| 59-1. | Saline | vaiPFC | 0.00537 |
| 59-1. | Saline | loPFC | 0.00507 |
| 59-2. | Saline | daiPFC | 0.00733 |
| 59-2. | Saline | vaiPFC | 0.00852 |
| 59-2. | Saline | loPFC | 0.00683 |

**Figure 4-1.** Data used to generate panel 4b.

| **Mouse** | **Age** | **Dopamine Varicosity Density (counts/um^3)** |
| --- | --- | --- |
| Adol1of4 | Adolescent | 0.00444 |
| Adol2of4 | Adolescent | 0.00443 |
| Adol3of4 | Adolescent | 0.00449 |
| Adol4of4 | Adolescent | 0.00413 |
| Ad3of6 | Adult | 0.00345 |
| Ad4of6 | Adult | 0.00306 |
| Ad5of6 | Adult | 0.00349 |
| Ad7of6 | Adult | 0.00326 |

**Figure 4-2.** Data used to generate panel 4c.

| **Mouse** | **Treatment** | **Dopamine Varicosity Density (counts/um^3)** |
| --- | --- | --- |
| 56-1. | Amphetamine | 0.00424 |
| 56-2. | Amphetamine | 0.00342 |
| 60-1. | Amphetamine | 0.00351 |
| 68-2. | Amphetamine | 0.00309 |
| 55-1. | Saline | 0.00460 |
| 55-2. | Saline | 0.00330 |
| 59-1. | Saline | 0.00400 |
| 59-2. | Saline | 0.00314 |
